# The Predictive Value of Serum Sodium Levels and Inflammatory Markers in Differentiating Complicated and Uncomplicated Acute Diverticulitis: A Retrospective Cohort Study

**DOI:** 10.3390/medicina61040592

**Published:** 2025-03-26

**Authors:** Bahadır Kartal, Mehmet Berksun Tutan, Veysel Barış Turhan, Furkan Uğur, Ertuğrul Gazi Alkurt

**Affiliations:** 1 Department of General Surgery, Erol Olçok Training and Research Hospital, 19040 Çorum, Turkey; 2 Department of General Surgery, Alaca State Hospital, 19600 Çorum, Turkey; mbtutan@gmail.com; 3 Department of General Surgery, Hitit University Faculty of Medicine, 19040 Çorum, Turkey; drbaristurhan@hotmail.com (V.B.T.); drfurkanugur@hotmail.com (F.U.); egalkurt@hotmail.com (E.G.A.)

**Keywords:** diverticulitis, complications, hyponatremia, prediction

## Abstract

*Background and Objectives*: This study aimed to investigate the role of serum sodium levels as an independent predictor of complications in acute diverticulitis and to evaluate their diagnostic value alongside inflammatory markers. *Materials and Methods*: A total of 134 patients diagnosed with acute diverticulitis between June 2018 and January 2024 at the Erol Olçok Training and Research Hospital were retrospectively analyzed. Complicated diverticulitis was defined based on the presence of an abscess, perforation, fistula, or obstruction classified as Hinchey stage II-IV. Serum sodium, CRP, and WBC levels were assessed for their predictive value. Statistical analyses included ROC analysis to determine optimal thresholds and logistic regression to evaluate independent predictors. *Results*: A total of 29.1% of the patients were classified as having complicated diverticulitis. Serum sodium levels were significantly lower in the complicated group (median: 133 mmol/L, *p* < 0.001), whereas CRP (median: 86.5 mg/L, *p* < 0.001) and WBC levels (median: 11.62 × 10^3^/µL, *p* = 0.001) were higher. The ROC analysis identified <135.5 mmol/L as the optimal threshold for serum sodium, with a 94.9% sensitivity and 94.7% specificity, making it the strongest predictor. The logistic regression revealed that each unit decrease in serum sodium increased the risk of complications by 5.7 times (*p* < 0.001). *Conclusions*: Serum sodium levels are an independent and strong predictor of complications in acute diverticulitis. When used alongside CRP and WBC levels, diagnostic accuracy can be enhanced, leading to improved patient management.

## 1. Introduction

Acute diverticulitis (AD) represents a significant burden in industrialized countries, with over 1.5 million inpatient treatment days and an annual hospital cost exceeding 2 billion dollars in the United States [1]. Diverticulosis, characterized by pouch-like protrusions in the colonic wall, affects approximately 40% of adults by age 60 and up to 80% of individuals over 85 in Western countries [2,3].

The treatment of diverticulitis depends on the clinical presentation and the presence of complications. Approximately 75–85% of diverticulitis episodes are uncomplicated, while the remainder involve complications such as an abscess, fistula, obstruction, or perforation [4,5]. The in-hospital mortality rate for diverticulitis ranges from 0.5% to 7%, but this rate increases to 17% in cases with abscesses and up to 45% in cases with perforations [6]. Complicated cases of diverticulitis, such as those with a perforation, are associated with significantly higher morbidity and mortality compared to simple cases. The Hinchey classification is widely used as a staging system to facilitate the clinical and radiological evaluation of diverticulitis. This classification enables the determination of appropriate treatment options, including conservative management, drainage, or surgical intervention, based on the severity of the clinical presentation [7]. While early stages in the Hinchey classification can be managed with antibiotics and minimally invasive techniques, Stage III and IV cases typically require emergency surgical intervention.

In cases of acute colonic diverticulitis, the Hinchey classification remains the most commonly used and easily recognized scale for assessing disease severity. However, the original Hinchey system relied on intraoperative findings for classification, preventing early-stage classification based on radiographic findings during the clinical course. The system has since been modified and validated for radiographic grading, and several other classification systems have been developed and validated to address the increasing reliance on computed tomography (CT) imaging. Despite the introduction of numerous scales designed to fill this gap, the utility of Hinchey-adapted scales remains limited, and newer systems have yet to achieve widespread clinical adoption.

C-reactive protein (CRP) is a well-established marker of infectious colonic perforation [8,9]. Hyponatremia (serum sodium level < 136 mmol/L) is the most common electrolyte disturbance and is associated with increased morbidity and mortality [10,11]. It has been evaluated as a significant biomarker, particularly in the presence of severe complications such as a perforation and abscess. The underlying mechanism involves the increased secretion of antidiuretic hormone (ADH) during inflammation, which enhances water retention and decreases sodium concentration. In the literature, the relationship between hyponatremia and various gastrointestinal diseases has been investigated, and it has been noted that sodium levels may also be affected in some benign conditions. However, studies directly examining the association between serum sodium levels and complications of acute diverticulitis are quite limited. Therefore, our study represents one of the few investigations contributing significantly to the literature on this topic. Pro-inflammatory cytokines, such as interleukin-6 and TNF-alpha, trigger ADH release, leading to hyponatremia. This mechanism is also linked to systemic inflammatory response syndrome (SIRS) and septic processes [12]. While hyponatremia is most commonly observed in pneumonia, it is also a recognized marker of spontaneous bacterial peritonitis in liver cirrhosis [13,14]. In addition to CRP and WBC, other inflammatory biomarkers such as the Neutrophil-to-Lymphocyte Ratio (NLR) and Platelet-to-Lymphocyte Ratio (PLR) have been studied for their potential role in differentiating complicated and uncomplicated cases in various inflammatory conditions. However, their clinical utility in acute diverticulitis remains under investigation.

In our study, the predictive value of plasma sodium concentration in distinguishing complicated from uncomplicated diverticulitis was investigated. Similar studies in adults are quite limited. Our study hypothesizes that plasma sodium concentration could serve as an alternative and/or auxiliary marker to the Hinchey classification for differentiating complicated from uncomplicated diverticulitis in adults.

## 2. Materials and Methods

Between June 2018 and January 2024, patients diagnosed with diverticulitis and hospitalized in the General Surgery Department at the Erol Olçok Training and Research Hospital were systematically assessed for inclusion in this study. Only patients with acute sigmoid diverticulitis confirmed by computed tomography (CT) were included in this study. Cases of diverticulitis involving other colonic segments were excluded to ensure a homogeneous cohort. Patients under the age of 18, those older than 85, individuals with known hematological, oncological, nephrological, or endocrinological diseases, patients with vascular or endothelial disorders, and those for whom any required study parameters were unavailable were excluded. A total of 162 patients diagnosed with acute diverticulitis were initially assessed for eligibility. After applying the exclusion criteria, a total of 28 patients were excluded: 12 patients due to age criteria (under 18 or over 85 years), 11 patients due to incomplete medical records, and 5 patients due to the use of steroids or diuretics that could interfere with sodium levels. Consequently, 134 patients met the inclusion criteria and were included in the final analysis.

Patients who were on steroid or diuretic therapy were excluded from the study to eliminate potential confounding effects on serum sodium levels. After applying these exclusion criteria, including the exclusion of patients on steroid or diuretic therapy, a total of 134 patients were included in the study. Among the demographic and clinical variables, ASA scores and BMI values were included for further analysis to assess their potential relationship with complications. Serum sodium levels were measured at the time of hospital admission and were used as baseline values in the analysis. Serum sodium levels were measured upon hospital admission, prior to the administration of any intravenous fluids, ensuring that the values reflect the patient’s initial physiological state. In addition to serum sodium, baseline vital parameters, including heart rate, blood pressure, respiratory rate, and body temperature, were recorded at admission. Furthermore, renal function markers (creatinine, blood urea nitrogen [BUN], estimated glomerular filtration rate [eGFR]), and serum osmolarity were assessed to evaluate their potential role in predicting disease severity.

This study was carried out in accordance with the 1964 Helsinki Declaration and its recent amendments. Ethical approval for the study was obtained from the Institutional Review Board of the Hitit University Faculty of Medicine Clinical Research Ethics Committee (Protocol Number: 2024/135). Patient consent was waived due to the retrospective and anonymized nature of the study.

A complication in this study was defined as the presence of diverticulitis associated with abscess formation, perforation, obstruction, or fistula development, classified as Hinchey stage II, III, or IV. Additional inclusion criteria included confirmed radiological evidence of diverticulitis. Exclusion criteria further encompassed patients who received antibiotics prior to hospital admission, those with incomplete medical records, or cases with concurrent infections or inflammatory conditions that could affect laboratory findings.

This study was conducted as a retrospective analysis. Statistical analyses were performed using IBM SPSS Statistics for Windows (version 26; IBM Corp., Armonk, NY, USA) and Minitab (version 22.2.1; Minitab, LLC, State College, PA, USA). Descriptive statistics for categorical variables were expressed as counts and percentages. For continuous variables, data with a normal distribution were presented as the mean ± standard deviation, while data not conforming to a normal distribution were reported as the median (minimum–maximum). The Shapiro–Wilk test was used to assess the normality of the data distribution.

Comparisons of numerical variables between independent groups were performed using the Student’s *t*-test for normally distributed data and the Mann–Whitney U test for non-normally distributed data. Categorical variables were compared using the Chi-square test. Correlations between variables were evaluated using Pearson or Spearman correlation coefficients, depending on the distribution of the data. Logistic regression analysis was utilized to assess interactions between variables and to determine the independent predictive value of serum sodium levels. A Receiver Operating Characteristic (ROC) curve analysis was employed to evaluate the discriminative ability of statistically significant variables. Cut-off values were determined using the area under the curve (AUC) and the Youden index. Sensitivity, specificity, positive predictive value (PPV), negative predictive value (NPV), and accuracy were calculated based on the cut-off points. Odds ratios (ORs) were computed for these cut-off values to quantify the strength of association. A *p*-value of <0.05 was considered statistically significant in all the analyses.

## 3. Results

A total of 134 patients were included in the study: 62 (46.27%) were male, and 72 (53.73%) were female. The cohort was divided into two groups: patients with complicated diverticulitis (*n* = 39) and those without complications (*n* = 95). The gender distribution between the two groups showed no statistically significant difference (*p* = 0.246). However, the mean age was significantly higher in the complicated group (61.05 ± 16.71 years) compared to the non-complicated group (54.58 ± 14.07 years, *p* = 0.024) (Table 1).

Baseline vital parameters were recorded at hospital admission, including heart rate, blood pressure, respiratory rate, and body temperature. The mean heart rate was 89 bpm, with no statistically significant difference between the groups (*p* = 0.127). Systolic and diastolic blood pressure values were also comparable between patients with and without complications (*p* = 0.278 and *p* = 0.312, respectively). Similarly, the respiratory rate (*p* = 0.184) and body temperature (*p* = 0.239) did not differ significantly between the groups. These findings suggest that vital parameters at admission were not strong predictors of complicated diverticulitis. The detailed values are summarized in Table 1.

Baseline renal function parameters and serum osmolarity levels were recorded upon hospital admission. The mean creatinine level was 0.89 mg/dL, with no statistically significant difference between the groups (*p* = 0.412). Similarly, blood urea nitrogen (BUN) and the estimated glomerular filtration rate (eGFR) did not differ significantly between the groups (*p* = 0.318 and *p* = 0.271, respectively). Additionally, serum osmolarity levels showed no significant variation between complicated and non-complicated cases (*p* = 0.345). These findings indicate that renal function and osmolarity were not significant predictors of complicated diverticulitis in our cohort. The detailed values are summarized in Table 1.

The hospitalization duration was similar between the two groups, with a median of 1 day in both groups (*p* = 0.098). Laboratory analyses revealed notable disparities between the groups. Serum sodium levels were significantly lower in the complicated group, with a median of 133 mmol/L, compared to 138 mmol/L in the non-complicated group (*p* < 0.001). Furthermore, CRP levels were substantially higher in patients with complications, with a median value of 86.5 mg/L, while the non-complicated group had a median of 19.1 mg/L (*p* < 0.001). White blood cell (WBC) counts were also significantly elevated in the complicated group, with a median of 11.62 × 10^3^/µL compared to 9.48 × 10^3^/µL in the non-complicated group (*p* = 0.001).

The assessment of disease severity using the mHinchey classification revealed distinct differences. While all the patients in the non-complicated group were classified as mHinchey 0 or 1a, the complicated group consisted of 79.49% mHinchey 1b and 20.51% mHinchey 2 cases (*p* < 0.001). A similar trend was observed with the Hinchey classification: all the patients in the non-complicated group were categorized as Hinchey 1, whereas in the complicated group, 79.49% were Hinchey 1 and 20.51% were Hinchey 2 (*p* < 0.001). Furthermore, a significant negative correlation was found between serum sodium levels and the mHinchey classification (Spearman’s ρ = −0.825, *p* < 0.001). This indicates that lower sodium levels were associated with more severe stages of diverticulitis. These findings suggest that hyponatremia may reflect the inflammatory burden and disease severity in acute diverticulitis.

There were no instances of mortality in either group (*p* = 1.000). However, surgical or interventional procedures were significantly more frequent in the complicated group, where three patients (7.69%) underwent intervention, compared to none in the non-complicated group (*p* < 0.001).

**Figure 1:**
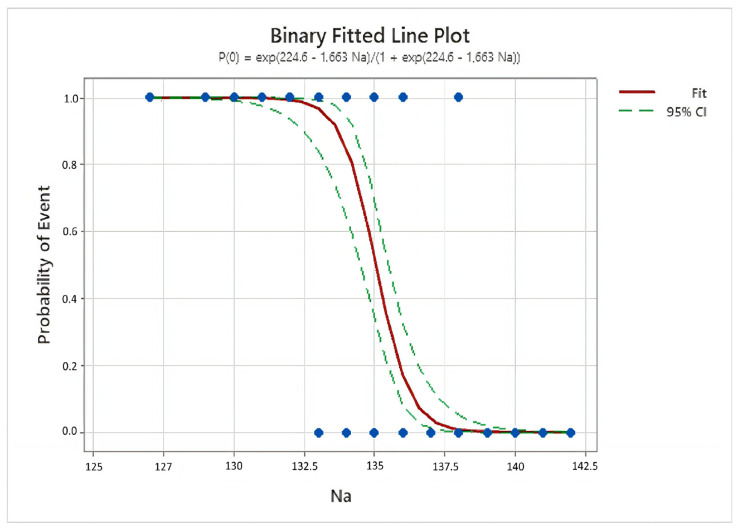
Binary logistic regression analysis result graph of serum sodium levels.

A multivariate logistic regression analysis was performed to determine if sodium levels were an independent predictor of complications in diverticulitis after controlling for confounders, such as gender and age. Gender was not found to be a significant predictor as it was also in the univariate analysis (*p* = 0.462). Although significant in the univariate analysis, age lost its significance and did not show statistical significance as a predictor in the multivariate analysis (*p* = 0.683). The binomial logistic regression model accurately classified 94.8% of the cases (Nagelkerke R^2^ = 0.808, *p* < 0.001) (Figure 1). Serum sodium levels were identified as a significant independent predictor of complications, even after adjusting for confounders (*p* < 0.001). For each unit decrease in Na levels, the odds of complications increased by approximately 5.7 times (ExpB: 5.675, 95% CI: 2.764–11.652, *p* < 0.001) (Table 1).

**Figure 2:**
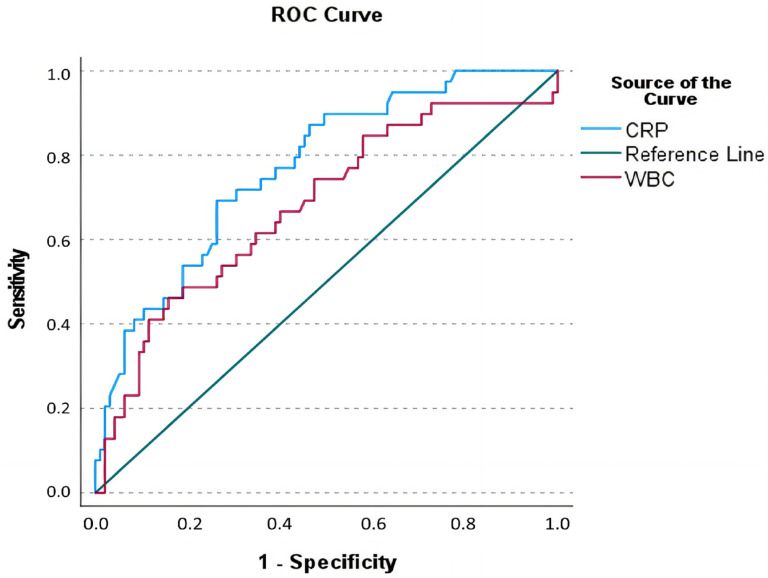
Receiver operating curve of C-reactive peptide levels and white blood cell count in the prediction of complicated diverticulitis.

To determine the optimal thresholds of serum sodium, CRP, and WBC count for predicting complications in diverticulitis, a ROC analysis was conducted (Figure 2). The optimal cut-off value for CRP was determined to be >65 mg/L, demonstrating a sensitivity of 69.2%, specificity of 73.7%, PPV of 51.9%, NPV of 85.4%, and a diagnostic accuracy of 72.39% (AUC: 0.768, SE: 0.043, 95% CI: 0.684–0.853, *p* < 0.001). For WBC, the optimal threshold was identified as >13.075 × 10^3^/µL, which provided a sensitivity of 46.2%, specificity of 84.2%, PPV of 54.5%, NPV of 79.2%, and a diagnostic accuracy of 73.13% (AUC: 0.678, SE: 0.053, 95% CI: 0.574–0.782, *p* = 0.001) (Table 2 and Figure 2).

**Figure 3:**
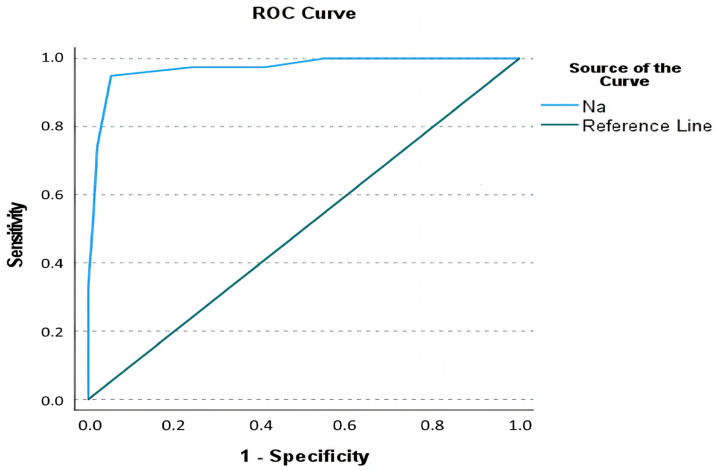
Receiver operating curve of serum sodium levels in the prediction of complicated diverticulitis.

For serum sodium, the most appropriate cut-off value was identified as <135.5 mmol/L, which yielded a sensitivity of 94.9%, specificity of 94.7%, PPV of 88.1%, NPV of 97.8%, and overall diagnostic accuracy of 94.78% (AUC: 0.972, SE: 0.015, 95% CI: 0.943–0.999, *p* < 0.001) (Figure 3 and Table 2). Sodium levels emerged as the strongest predictor of complications, with values below 135.5 mmol/L increasing the odds of complications by approximately 332 times (OR: 333, 95% CI: 61.821–1793.716, *p* < 0.001).

To improve the diagnostic performance of Na, CRP, and WBC levels, we evaluated rule-in and rule-out cut-off values. The rule-out cut-offs, chosen to maximize sensitivity, were <137.5 mmol/L for Na (98.7% sensitivity), >40 mg/L for CRP (96.1% sensitivity), and >9.8 × 10^3^/µL for WBC (93.2% sensitivity). Conversely, the rule-in cut-offs, selected for high specificity to confirm complications, were <133.5 mmol/L for Na (95.6% specificity), >90 mg/L for CRP (92.7% specificity), and >13.2 × 10^3^/µL for WBC (91.9% specificity). These findings are summarized in Table 3.

## 4. Discussion

The accurate assessment of the clinical presentation and early detection of complications are crucial in the treatment of diverticulitis. Historically, diverticulitis has been thought to progress to complications such as an abscess, fistula, or perforation; however, the role of biomarkers used in this process is increasingly being investigated [15]. In our study, we evaluated the predictive value of baseline serum sodium levels, along with inflammatory markers such as WBC count and CRP levels, in differentiating complicated from uncomplicated diverticulitis in patients hospitalized and followed up for acute diverticulitis.

Our findings demonstrated that serum sodium levels are a strong independent predictor of complications, with a significantly increased risk of complications observed as sodium levels decrease. Additionally, CRP and WBC levels showed significant differences between the complicated and uncomplicated groups and exhibited notable predictive performance in ROC analyses.

Studies investigating inflammatory markers such as CRP from blood samples in the diagnosis of acute diverticulitis and the identification of complications have reported varying results and threshold values. In the study by Nizri et al., a CRP level of <50 mg/L was identified as a reliable threshold for excluding complicated disease, which could help reduce unnecessary CT scans [16]. However, it was concluded that the diagnostic value of CRP is limited in patients using corticosteroids, highlighting the need for alternative biomarkers for this patient group.

Conversely, other studies have emphasized that CRP can be used as an effective marker for predicting the severity of acute diverticulitis, recommending mandatory CT imaging in patients with CRP levels ≥ 150 mg/L [17]. For CRP levels between 50–150 mg/L, the necessity of CT imaging should be evaluated based on the patient’s clinical condition.

In our study, CRP emerged as a significant biomarker for predicting complications in patients with acute diverticulitis. We found that CRP levels were significantly higher in the complicated diverticulitis group, with a mean value of 86.5 mg/L. The ROC analysis revealed that a threshold of >65 mg/L provided diagnostic performance with a sensitivity of 69.2% and a specificity of 73.7%. However, the relatively low positive predictive value (51.9%) suggests that CRP alone may not be sufficient for assessing the risk of complications and should be complemented with other markers, such as sodium levels or WBC counts.

Although WBC count is often observed to be elevated in acute diverticulitis, its diagnostic value in distinguishing complicated from uncomplicated cases is limited. While studies have reported significantly higher WBC levels in patients with complicated diverticulitis, the specificity and sensitivity of this marker remain lower compared to other inflammatory markers, such as CRP [18,19]. Notably, an increase in WBC levels raises the suspicion of complicated diverticulitis; however, diagnosis based solely on this parameter is generally not feasible.

Thus, the limited diagnostic value of WBC underscores the need for its evaluation alongside more reliable markers like CRP and radiological imaging. In a study by van de Wall et al., WBC was found inadequate for differentiating between complicated and uncomplicated diverticulitis, whereas CRP emerged as a more effective marker for identifying complications [15]. Similarly, Kumarasinghe et al. emphasized that although WBC levels are elevated in patients with complicated diverticulitis, its diagnostic accuracy is inferior to CRP [19]. These findings highlight the importance of a multidisciplinary approach in the diagnosis of acute diverticulitis.

In our study, although WBC levels showed a significant difference in predicting complicated diverticulitis, its sensitivity was low, indicating that it cannot be considered a sufficient standalone marker. Beyond CRP and WBC, alternative inflammatory markers such as NLR and PLR have been investigated in various inflammatory diseases. However, these parameters were not included in our study due to the primary focus on widely used, well-established, and easily accessible biomarkers in clinical practice. Future studies integrating a broader range of biomarkers may provide additional insights into their diagnostic utility in acute diverticulitis.

Although biomarkers such as Procalcitonin (PCT) have been extensively studied in sepsis and systemic inflammatory response syndrome (SIRS), their role in acute diverticulitis remains unclear. PCT is not routinely used in clinical practice for diverticulitis, and its diagnostic utility in differentiating complicated and uncomplicated cases has not been well established. Additionally, while other electrolytes such as potassium and calcium could also be analyzed, sodium remains the most commonly associated parameter with inflammatory processes and systemic disease severity. Future studies incorporating a broader range of biomarkers may provide additional insights.

The Hinchey classification has been a cornerstone in determining the severity of acute diverticulitis cases for many years. Its ability to categorize diverticulitis with perforation into four distinct stages contributes significantly to guiding surgical and medical treatment options. However, the limitations of this classification are well-documented, and it is frequently emphasized that it is not a sufficient standalone tool for diagnostic decision-making.

In a systematic review by Cirocchi et al., outcomes of different surgical methods for patients in Hinchey stages III and IV were compared, revealing that the classification might lead to heterogeneous results in clinical decision-making [20]. This limitation stems from the classification’s reliance solely on intraoperative or radiological findings, providing limited information in the early stages of clinical progression. Similarly, Ahmadi’s study highlighted the inadequacy of the Hinchey classification in predicting patient responses to conservative treatment [21]. The study suggested that integrating CRP and other inflammatory markers, particularly in cases with complications such as abscess or peritonitis, could enhance its predictive power.

Although the correlation between the Hinchey classification and CRP levels indicates that higher inflammatory stimulation often aligns with higher Hinchey stages, this relationship falls short of providing dynamic monitoring of the inflammatory process. Our study demonstrated a strong negative correlation between serum sodium levels and the mHinchey classification (Spearman’s ρ = −0.825, *p* < 0.001), suggesting that hyponatremia is more prevalent in patients with advanced stages of diverticulitis. This aligns with the hypothesis that systemic inflammatory response and excessive ADH secretion contribute to sodium imbalance in severe cases. Future studies may further explore the predictive value of sodium levels in guiding treatment strategies.

Additionally, Choi et al. underscored the superior predictive accuracy of alternative systems, such as the AAST (American Association for the Surgery of Trauma) classification, compared to Hinchey [22]. The AAST classification’s ability to evaluate a broader set of clinical variables offers a distinct advantage in assessing the severity of complications and guiding clinical decisions.

In light of this evidence, it is apparent that the Hinchey classification should be complemented by more robust tools, particularly biomarkers or modern classification systems, to improve the early identification of patients at risk of complications and to guide treatment strategies. This combined approach could enhance diagnostic accuracy and optimize patient management. Based on the findings of this study, while the Hinchey classification remains a valuable guide for evaluating complicated diverticulitis, its diagnostic accuracy can be further enhanced when used alongside inflammatory markers such as sodium, CRP, and WBC. This integrated strategy offers a more comprehensive approach to patient care.

Although the etiology of hyponatremia in complicated acute diverticulitis is not fully understood, it is likely mediated by the antidiuretic hormone (ADH) [23]. Some studies have demonstrated that hyponatremia serves as a predictive marker for cholecystitis and colonic perforation in adult patients [24,25]. However, data on the association between hyponatremia and complicated diverticulitis in adults are extremely limited. During our investigation, only a few studies addressing this topic were identified. For instance, Käser et al. found that hyponatremia (serum sodium < 136 mmol/L) was a specific marker for infected colonic perforation in patients over 50 years of age [26]. However, their study noted that the sensitivity of hyponatremia (31%) was low, indicating that it is not a sufficient standalone marker for predicting perforation. Nevertheless, the study highlighted that low-cost and easily measurable parameters, such as hyponatremia, can enhance diagnostic accuracy when used alongside other inflammatory markers like CRP.

Due to the limited number of cases requiring surgical or interventional procedures, a detailed subgroup analysis could not be performed. However, future prospective studies with larger sample sizes should aim to evaluate whether hyponatremia and other inflammatory markers differ between these subgroups.

Hyponatremia is gaining importance as a potential marker for complications in acute diverticulitis and other abdominal inflammatory conditions. Its pathophysiology involves the nonosmotic release of ADH, triggered by inflammatory cytokines such as interleukin-6. In this study, it was not possible to distinguish between hypovolemic, normovolemic, and hypervolemic hyponatremia due to the retrospective nature of the data. However, the mechanism of hyponatremia in complicated diverticulitis is likely related to the non-osmotic release of antidiuretic hormone (ADH), triggered by inflammatory cytokines such as interleukin-6. This process leads to water retention and dilutional hyponatremia, which are common in severe inflammatory conditions. This mechanism leads to water retention and dilutional hyponatremia, particularly evident in severe inflammatory conditions such as perforation. While studies on appendicitis and other abdominal conditions have reported varying results regarding the diagnostic value of hyponatremia, its significance as an indicator of disease severity is consistent. For example, Messias et al. reported that hyponatremia (serum sodium < 136 mEq/L) increased the likelihood of complicated appendicitis fivefold [27]. Similarly, Lindestam et al. identified a strong association between low plasma sodium concentration and the risk of perforation in children, with sodium levels below 136 mmol/L increasing the likelihood of perforation 32-fold [28].

In the context of acute diverticulitis, these findings align with our study, which demonstrated a significant association between hyponatremia and disease severity. The low serum sodium levels observed in complicated cases underscore the inflammatory and systemic effects of severe diverticular disease. This suggests that serum sodium, when combined with other inflammatory markers such as CRP and WBC, can serve as a cost-effective and easily accessible diagnostic tool, particularly in resource-limited healthcare settings. However, the diagnostic sensitivity and specificity of sodium levels are influenced by individual variability and disease heterogeneity. Therefore, hyponatremia should be interpreted alongside clinical evaluation and radiological findings to optimize decision-making in diverticulitis management.

This study represents a retrospective analysis evaluating the value of serum sodium levels and inflammatory markers in predicting complications in acute diverticulitis. The strengths of our study include a logistic regression model with high diagnostic accuracy (Nagelkerke R^2^ = 0.808), the determination of optimal thresholds through ROC analyses, and the inclusion of a large patient cohort. Additionally, the use of widely accessible parameters such as CRP, WBC, and sodium enhances the study’s practical clinical applicability. However, the retrospective design carries potential risks of selection bias and missing data. Due to the retrospective nature of this study, individual symptoms such as diarrhea were not systematically documented, and no formal sepsis scoring system (e.g., SOFA, NEWS2) was applied at the time of hospital admission. However, it is well established that acute diverticulitis commonly presents with symptoms such as abdominal pain, fever, and altered bowel habits, including both constipation and diarrhea. Additionally, patients presenting with septic shock or clinically significant sepsis were excluded from the study to maintain a homogeneous cohort. Future prospective studies incorporating a more comprehensive assessment of symptoms and standardized sepsis scoring systems may provide additional insights into their diagnostic and prognostic significance.

The relatively low rate of surgical intervention observed in this study can be attributed to the retrospective and single-center nature of data collection. The patient population at our center primarily consisted of individuals who were managed conservatively, which may not fully represent the natural course of the disease. Additionally, referral patterns, local practice preferences, and resource availability could have influenced the lower surgical intervention rates in this cohort. Moreover, the generalizability of the findings may be limited due to the broad exclusion criteria. A significant limitation of this study is the inability to classify the types of hyponatremia (hypovolemic, normovolemic, or hypervolemic), which would have provided a more detailed understanding of the underlying mechanisms. Prospective studies are required to validate these results further.

## 5. Conclusions

This study demonstrates that serum sodium levels are an independent and strong predictor of complications in patients with acute diverticulitis. Alongside sodium, CRP and WBC levels provide valuable insights for clinical assessment. In particular, serum sodium levels below 135.5 mmol/L were significantly associated with an increased risk of complications. These findings highlight the importance of integrating biomarkers with clinical evaluation and imaging techniques to enhance diagnostic accuracy and improve patient management.

## Figures and Tables

**Table 1:** General characteristics of the patients and results of univariate and multivariate analyses.

Variables		All Patients (*n* = 134)	Complicated (*n* = 39)	No Complication (*n* = 95)	*p*	Wald	ExpB (95% CI)	*p*
Gender	Male	62 (46.27%)	15 (38.46%)	47 (49.47%)	0.246	0.541	1.771 (0.386–8.131)	0.462
	Female	72 (53.73%)	24 (61.54%)	48 (50.53%)				
Age		56.46 ± 15.11	61.05 ± 16.71	54.58 ± 14.07	0.024	0.167	1.011 (0.959–1.066)	0.683
Hospitalization Duration		1 (1–12)	1 (1–12)	1 (1–11)	0.098			
ASA Score		2.3 ± 0.6	2.3 ± 0.4	2.2 ± 0.5	0.320			
BMI (kg/m^2^)		25.4 ± 4.2	26.1 ± 4.5	25.0 ± 3.9	0.084			
Na		137 (127–142)	133 (127–138)	138 (133–142)	<0.001	22.373	5.675 (2.764–11.652)	<0.001
CRP		39,12 (3–267)	86.5 (4.88–267)	19.1 (3–212)	<0.001			
WBC		10.02 (3.76–37.4)	11.62 (3.76–22.96)	9.48 (4.5–37.4)	0.001			
mHinchey	0	6 (4.48%)	0 (0.00%)	6 (6.32%)	<0.001			
	1a	89 (66.42%)	0 (0.00%)	89 (93.68%)				
	1b	31 (23.13%)	31 (79.49%)	0 (0.00%)				
	2	8 (5.97%)	8 (20.51%)	0 (0.00%)				
Hinchey	1	126 (94.03%)	31 (79.49%)	95 (100.00%)	<0.001			
	2	8 (5.97%)	8 (20.51%)	0 (0.00%)				
Mortality		0 (0.00%)	0 (0.00%)	0 (0.00%)	1.000			
Operation/Intervention		3 (2.24%)	3 (7.69%)	0 (0.00%)	<0.001			
Heart Rate (bpm)		89 ± 14	91 ± 15	88 ± 13	0.127			
Systolic BP (mmHg)		126 ± 18	124 ± 19	127 ± 17	0.278			
Diastolic BP (mmHg)		78 ± 12	76 ± 13	79 ± 11	0.312			
Respiratory Rate		19 ± 3	20 ± 3	19 ± 3	0.184			
Temperature (°C)		37.4 ± 0.6	37.5 ± 0.7	37.3 ± 0.5	0.239			
Creatinine (mg/dL)		0.89 ± 0.22	0.91 ± 0.24	0.91 ± 0.24	0.412			
BUN (mg/dL)		15.8 ± 4.6	16.2 ± 5.1	15.6 ± 4.3	0.318			
eGFR (mL/min/1.73 m^2^)		94 ± 18	91 ± 19	96 ± 17	0.271			
Serum Osmolarity (mOsm/kg)		290 ± 5	289 ± 6	291 ± 5	0.345			

CI: confidence interval, Na: serum sodium level, CRP: C-reactive peptide, WBC: white blood cell count, and mHinchey: modified Hinchey.

**Table 2:** Diagnostic values of optimal cut-off points for variables and results of ROC analysis.

Variables	Cut-Off	Diagnostic Values					ROC Analysis			Odds Ratio		
		Sensitivity	Specificity	PPV	NPV	Accuracy	Area (SE)	95%CI	*p*	Odds Ratio	95%CI	*p*
Na	<135.5	94.9%	94.7%	88.1%	97.8%	94.78%	0.972 (0.015)	0.943–0.999	<0.001	333	61.821–1793.716	<0.001
CRP	>65	69.2%	73.7%	51.9%	85.4%	72.39%	0.768 (0.043)	0.684–0.853	<0.001	45.357	2.777–14.291	<0.001
WBC	>13.075	46.2%	84.2%	54.5%	79.2%	73.13%	0.678 (0.053)	0.574–0.782	0.001	4.571	1.980–10.557	<0.001

PPV: positive predictive value, NPV: negative predictive value, ROC: receiver operating curve, SE: standard error, CI: confidence interval, *p*: statistical significance, and Na: serum sodium levels.

**Table 3:** Rule-In and Rule-Out Cut-Off Values for Na, CRP, and WBC.

Variables	Rule-Out Cut-Off (High Sensitivity)	Sensitivity	Specificity	Rule-In Cut-Off (High Specificity)	Sensitivity	Specificity
Na (mmol/L)	<137.5 mmol/L	98.7%	81.3%	<133.5 mmol/L	72.4%	95.6%
CRP (mg/L)	>40 mg/L	96.1%	65.4%	>90 mg/L	61.3%	92.7%
WBC (×10^3^/µL)	>9.8 × 10^3^/µL	93.2%	68.5%	>13.2 × 10^3^/µL	57.4%	91.9%

Na: sodium, CRP: C-reactive protein, and WBC: white blood cell count.

## Data Availability

Data are available from Bahadır Kartal upon reasonable request due to the privacy policies of the institute.

## Abbreviations

The following abbreviations are used in this manuscript:

AD	Acute diverticulitis
ADH	Antidiuretic hormone
AUC	Area under the curve
ASA	American Society of Anesthesiologists (Score)
AAST	American Association for the Surgery of Trauma
BMI	Body mass index
CI	Confidence interval
CRP	C-reactive protein
CT	Computed tomography
mHinchey	Modified Hinchey (classification)
Na	Serum sodium level
NPV	Negative predictive value
OR	Odds ratio
PPV	Positive predictive value
ROC	Receiver Operating Characteristic
SIRS	Systemic inflammatory response syndrome
TNF-alpha	Tumor Necrosis Factor Alpha
WBC	White blood cell count
SE	Standard error
SPSS	Statistical Package for the Social Sciences
